# Connecting paths between juvenile and adult habitats in the Atlantic green turtle using genetics and satellite tracking

**DOI:** 10.1002/ece3.4708

**Published:** 2018-12-11

**Authors:** Philippine Chambault, Benoît de Thoisy, Maïlis Huguin, Jordan Martin, Marc Bonola, Denis Etienne, Julie Gresser, Gaëlle Hiélard, Julien Mailles, Fabien Védie, Cyrille Barnerias, Emmanuel Sutter, Blandine Guillemot, Émilie Dumont‐Dayot, Sidney Régis, Nicolas Lecerf, Fabien Lefebvre, Cédric Frouin, Nathalie Aubert, Christelle Guimera, Robinson Bordes, Laurent Thieulle, Matthieu Duru, Myriam Bouaziz, Adrien Pinson, Frédéric Flora, Patrick Queneherve, Thierry Woignier, Jean‐Pierre Allenou, Nicolas Cimiterra, Abdelwahab Benhalilou, Céline Murgale, Thomas Maillet, Luc Rangon, Noémie Chanteux, Bénédicte Chanteur, Christelle Béranger, Yvon Le Maho, Odile Petit, Damien Chevallier

**Affiliations:** ^1^ CNRS‐IPHC UMR 7178 Université de Strasbourg Strasbourg Cedex 2 France; ^2^ Association Kwata Cayenne Cedex France; ^3^ DEAL Martinique, Pointe de Jaham Schoelcher Cedex France; ^4^ Office de l'Eau Martinique Fort‐de‐France France; ^5^ Section Boisbert Délégation Inter Régionale Outre‐mer, Chemin de Boyer Lamentin France; ^6^ Surfrider Foundation Europe Martinique France; ^7^ IRD Martinique‐Caraïbe Le Lamentin Cédex 2 France; ^8^ CNRS, IRD, IMBE Aix Marseille University, University Avignon Marseille France; ^9^ IFREMER Délégation de Martinique Le Robert France; ^10^ Association POEMM Les Trois Ilets France; ^11^ PNR Martinique Fort‐de‐France France; ^12^ CNRS Équipe d'Éthologie Cognitive et Sociale Strasbourg France

**Keywords:** *Chelonia mydas*, developmental habitats, immature green turtle, migration routes, mixed stock analysis

## Abstract

Although it is commonly assumed that female sea turtles always return to the beach they hatched, the pathways they use during the years preceding their first reproduction and their natal origins are most often unknown, as it is the case for juvenile green turtles found in Martinique waters in the Caribbean. Given the oceanic circulation of the Guiana current flowing toward Martinique and the presence of important nesting sites for this species in Suriname and French Guiana, we may assume that a large proportion of the juvenile green turtles found in Martinique are originating from the Suriname–French Guiana beaches. To confirm this hypothesis, we performed mixed stock analysis (MSA) on 40 green turtles sampled in Martinique Island and satellite tracked 31 juvenile green turtles tagged in Martinique to (a) assess their natal origin and (b) identify their destination. Our results from MSA confirm that these juveniles are descendant from females laying on several Caribbean and Atlantic beaches, mostly from Suriname and French Guiana, but also from more southern Brazilian beaches. These results were confirmed by the tracking data as the 10 turtles leaving Martinique headed across the Caribbean–Atlantic region in six different directions and 50% of these turtles reached the Brazilian foraging grounds used by the adult green turtles coming from French Guiana. One turtle left the French Guianan coast to perform the first transatlantic migration ever recorded in juvenile green turtles, swimming toward Guinea‐Bissau, which is the most important nesting site for green turtles along the African coast. The extensive movements of the migrant turtles evidenced the crossing of international waters and more than 25 exclusive economic zones, reinforcing the need for an international cooperative network to ensure the conservation of future breeders in this endangered species.

## 1 INTRODUCTION

Although the migratory behavior has evolved as a strategy to maximize fitness in response to a seasonal environment, long‐distance migrations depend on both biotic (e.g., competition, predation, reproduction) and abiotic (e.g., seasonality) factors (Alerstam, Hedenström, & Åkesson, 2003). Migration can therefore be triggered by the search for food, a shelter, or a mate (Dingle & Drake, 2007). A favorable habitat for survival, accordingly to perform a foraging activity, will not necessarily be a favorable habitat for reproduction, and conversely. That is the reason why many species perform long‐distance migrations, exploiting distinct habitats during their life cycle. In the marine realm, such long migrations have been reported in seabirds (e.g., Egevang et al., 2010; Storr, 1958), cetaceans (e.g., Fossette et al., 2014; Garrigue et al., 2010; Rasmussen et al., 2007), pinnipeds (e.g., Hindell et al., 2016; Robinson et al., 2012), large pelagic fishes (e.g., Block et al., 2001; Potter, Galuardi, & Howell, 2011; Skomal et al., 2009), and sea turtles (e.g., Chambault et al., 2015; Chambault, de Thoisy, Heerah, et al., 2016; Chambault et al., 2017; Polovina et al., 2004).

The migration of sea turtles is directly related to their complex life cycle which encompasses neritic, marine and/or oceanic habitats. Except the flatback and the leatherback turtles that adopt a particular life history pattern (exclusively neritic and exclusively oceanic, respectively), the five remaining species alternate between neritic and oceanic habitats during their life cycle (Bolten, 2003). After passively drifting for several years in oceanic environments, that is, the “lost years” (Putman & Naro‐Maciel, 2013; Reich, Bjorndal, & Bolten, 2007), juvenile turtles then recruit to shallow neritic habitats (Musick & Limpus, 1997). They thereafter spend many years foraging in these developmental habitats (Tomaszewicz et al., 2015) until they reach sexual maturity before migrating back to their adult habitats, either nesting site or foraging grounds (Bjorndal, Bolten, & Chaloupka, 2005; Patrício, Velez‐Zuazo, Diez, Van Dam, & Sabat, 2011).

To elucidate the mystery of the “lost years,” many studies have used numerical models of ocean circulation to model the dispersal of juvenile sea turtles during the oceanic stage (Blumenthal et al., 2009; Hays, Fossette, Katselidis, Mariani, & Schofield, 2010; Mansfield & Putman, 2013; Monzón‐Argüello et al., 2010; Proietti et al., 2012). These studies were firstly based on the assumption that juvenile turtles are passively drifted by ocean currents during their oceanic stage, that is, the “passive drift” theory (Carr & Meylan, 1980). But some recent studies have suggested that, rather than a passive dispersal, the movements of juvenile sea turtles are based on a directed swimming behavior (the “active theory”) (Briscoe et al., 2016; Gaspar & Lalire, 2017, 2012; Hamann, Grech, Wolanski, & Lambrechts, 2011; Putman & Mansfield, 2015; Putman & Naro‐Maciel, 2013; Putman, Scott, Verley, Marsh, & Hays, 2012; Putman, Verley, Shay, & Lohmann, 2012; Scott, Marsh, & Hays, 2012).

Given their long‐lived, slow‐growing, migratory behavior and their late sexual maturity (e.g., between 15–50 years for the green turtle, Avens & Snover, 2013; Bell et al., 2005; Bjorndal, Bolten, & Martins, 2000; Patricio, Diez, & van Dam, 2014), some parts of the life cycle of sea turtles are still poorly understood, especially after leaving their developmental habitat. To fill this gap, genetic approaches relying on a maternally inherited gene, such as the cytochrome b, may be used to assess the composition of a population of juvenile turtles at their feeding grounds, and retrace the connections between juvenile and adult habitats (Bowen & Karl, 2007; Hatase et al., 2002). The ability to distinguish nesting aggregations based on haplotype frequencies has clarified dispersal patterns of hatchlings and juveniles (Bjorndal, Bolten, Moreira, Bellini, & Marcovaldi, 2006; Bowen & Karl, 2007). Feeding grounds are visited by individuals from different natal origins, constituting mixed stocks (Bowen & Karl, 1997). mixed stock analysis (MSA) thus enables investigating the contribution of each source, that is, a nesting site, to a feeding ground, based on the significant differences of haplotype frequencies (Bolker, Okuyama, Bjorndal, & Bolten, 2003, 2007; Okuyama & Bolker, 2005). This technique has proved to be useful for multiple migratory vertebrates such as fishes (Bradbury et al., 2016; Dunton et al., 2012; Ruzzante, Taggart, Lang, & Cook, 2000), whales (Albertson‐Gibb et al., 2008; Schmitt et al., 2014), and sea turtles (Bowen et al., 2007; Jordao, Bondioli, Guebert, Thoisy, & Almeida‐Toledo, 2015; Naro‐Maciel et al., 2012, 2014; Proietti et al., 2012; Prosdocimi, Dutton, Albareda, & Remis, 2014; Prosdocimi, González Carman, Albareda, & Remis, 2012; Vilaça, Lara‐Ruiz, Marcovaldi, Soares, & Santos, 2013).

Based on the assumption that female sea turtles always return on the beach they hatched (Meylan, 1999), genetic studies of juveniles may contribute to decipher their future adult habitats. In the Western Atlantic, several studies have been conducted to assess the natal origin of juvenile green turtles and they have shown a strong genetic diversity, especially within the Caribbean (Bass & Witzell, 2000; Bass, Epperly, & Braun‐McNeill, 2006; Lahanas et al., 1998; Luke, Horrocks, LeRoux, & Dutton, 2003). Yet, the pathways juvenile green turtles use during the years preceding their first reproduction and their natal origins are unknown, as it is the case for the immature green turtles found in Martinique waters in the Caribbean. In Martinique Island, the green turtle was overexploited for its flesh and its eggs for 500 years (Chevalier, 2006), leading to the disappearance of nesting females on this island. However, many juvenile green turtles are found year‐round in this habitat (Chevalier, 2006), likely due to the high abundance of seagrass (in comparison with the lack of seagrass in French Guianese waters) and a protection from predators (Chevallier et al., 2016). But to date, no information on their natal origin and developmental migrations is available.

Given the oceanic circulation of the Guiana current flowing toward Martinique (Borstad, 1982) and the presence of important nesting sites for green turtles in Suriname and French Guiana (Chambault, de Thoisy, Kelle, et al., 2016), we assume that a large proportion of the juvenile green turtles found in Martinique are originating from the Suriname–French Guiana beaches. To confirm this hypothesis, we performed MSA on 40 green turtles sampled in Martinique Island and satellite tracked 31 juvenile green turtles from Martinique to (a) assess their natal origin and (b) identify their destination during developmental migrations. These two complementary techniques provide novel information on the connecting paths between the juvenile habitat of Martinique and the adult habitats across the Atlantic. By identifying the pathways used by juvenile green turtles, the present study can also provide a solid basis for an adequate conservation of this endangered species by protecting future breeders at the Caribbean–Atlantic scale.

## 2 METHODS

### 2.1 Animal capture and tagging

The fieldwork was conducted randomly in the Anses d'Arlet, in the waters of Martinique Island (14°30ʹ9.64ʺN, 61°5ʹ11.85ʺW, French West Indies, France) at depth up to 15 m. Between 2013 and 2017 (from June to October of each year), 425 juvenile green turtles were captured by teams composed of three free divers. After spotting a static turtle feeding or resting at the bottom, a free diver would dive close to the head of the turtle, as discreetly as possible in order to avoid detection. Once close enough, the free diver would catch the turtle by the pygales plates of the shell (located behind the nuchal), and bring it to the surface. A second diver would then hold the foreflippers and help lift the individual into the boat for measurements and tagging. Each turtle was placed in a pen, and standard morphometric data recorded, that is, curved carapace length (CCL) and body mass using an electronic dynamometer. Each individual was marked by inserting a Passive Integrated Transponder (PIT) tag in the right triceps.

Between October 2013 and October 2015, 11 Argos‐Fastloc GPS tag (Wildlife Computers Redmond, WA, USA) and 9 GPS‐Satellite Relayed Data Loggers (GPS‐SRDL, Sea Mammal Research Unit, University of St. Andrews, Scotland) were fixed on juvenile green turtles using epoxy resin following Baudouin et al.’s method (2015). Using the same procedure, between October 2016 and October 2017, 11 individuals were equipped with SPOT tags (Wildlife Computers Redmond, WA, USA).

### 2.2 Field sampling

Skin samples were collected from 425 juvenile green turtles captured in Martinique by the free divers from 2013 to 2016, and 40 samples were used for genetic analysis. All skin samples were collected on the anterior flipper of individuals using a biopunch (Biopsy Punch BioPunch Dermal OR Grade) with a diameter of 4 or 6 mm (depending of the size of the individuals). To avoid resampling, a PIT was first checked on each individual. Samples were then conserved in absolute ethanol and maintained at −20°C until DNA extraction.

### 2.3 Genetic analysis

The use of the genetic resources was declared to the French Ministry of Environment under the reference TSP 79.585, in compliance with the Access and Benefit Sharing procedure implemented by the Loi pour la Reconquête de la Biodiversité.

#### 2.3.1 DNA extraction and D‐loop control region genotyping

Biopsy punches were prelysed within the EasyMAG lysis buffer (BioMérieux, Marcy l'Etoile, France) at 4°C overnight, then in Tris–SDS buffer and proteinase K at 56°C before grinding. DNA was isolated using NucliSENS EasyMAG® bio‐robot (BioMérieux, Marcy l'Etoile, France) following standard protocols for tissue. The DNA pellet extracted was resuspended in H2O PPI and stored at −20°C until use. The primers LCM15382 and H950 (Abreu‐Grobois et al., 2006) were used to amplify the mitochondrial DNA (mtDNA) control region (D‐loop) of 118 individuals. PCRs were performed using a standard procedure with the BIOTAQ™ DNA Polymerase PCR kit (BioLine Reagents Limited, London, UK). PCR was carried out in a 48µl PCR containing 2 µl of genomic DNA, 5µl 10 × buffer, 3 µl of MgCl_2_, 5 µl of dNTP, 4 µl of each primer, and 0.5 µl of BIOTAQ™ DNA Polymerase (BioLine, London, UK). The cycling conditions included an initial denaturation step at 95°C for 5 min followed by 36 denaturation cycles at 94°C for 30 s, annealing for 30 s at 50°C, and elongation at 72°C for 30 s. A final 10 min extension step at 72°C followed the last cycle. Beckman Coulter Genomics carried out sequencing (Beckman Coulter Genomics, Takeley, UK).

#### 2.3.2 MtDNA D‐loop sequence analysis

DNA sequences generated for Martinique samples were edited and aligned with Mega 6.06 (Tamura, Stecher, Peterson, Filipski, & Kumar, 2013). Genetic signatures were compared to Atlantic nesting rookeries used and published previously (Jordao et al., 2017). Gene diversity (Hs), nucleotide diversity (*π*), and genetic differentiation (FST averaged over loci, Weir & Cockerham, 1984) were obtained for each population from Arlequin 3.5 (Excoffier, Smouse, & Quattro, 1992) with 99,999 permutations. Haplotype distribution was established using DNASP 5.10.1 (Rozas, 2009; Rozas & Rozas, 1999), and haplotype networks were constructed using the median joining network algorithm implemented in Network 5.0 (Bandelt, Forster, & Röhl, 1999).

Mixed stock analyses were used to evaluate the contribution of each nesting population to the juvenile green sea turtles sampled in Martinique, using all described Atlantic nesting rookeries genetic profiles (Jordao et al., 2017). These analyses follow a Bayesian approach, estimating the contribution of different source populations (nesting sites) to a mixed population (Bolker, Okuyama, Bjorndal, & Bolten, 2007). Haplotypes found at feeding grounds, which cannot be tracked back to the source population (“orphan” haplotypes), were excluded from the analyses. In the same way, haplotypes found at nesting sites but not at feeding ground of Martinique were excluded from the analyses.

The many‐to‐one Bayesian approach of mixed stock analyses has used and evaluated the contribution of each nesting population to Martinique population by considering that each nesting population analyzed would have the same probability to contribute to the mixed stock. The Gelman and Rubin (1992) diagnostic has been used to test the convergence strength of chains. Estimated shrink factors close to one indicate convergence, and acceptable values are less than 1.2 (Kass, Carlin, Gelman, & Neal, 1998).

### 2.4 Tracking data analysis

Data were downloaded daily via Argos Message Retriever (WC‐DAP, Wildlife Computers‐Data Analysis Programs). In order to provide optimum location accuracy and increase the number of positions available to counterbalance errors caused by the proximity to the shore, the GPS‐SRDL tags were programmed to simultaneously record Argos and GPS locations. The GPS sampling interval was set to 15 min. Tag position estimates (Argos data) were enhanced by applying a Kalman‐filtering algorithm to account for Argos error (CLS, Collecte Localisation Satellites, Toulouse, France).

The proximity to the shore and the possible Argos error resulted in 51% of the positions being found on land after applying the Kalman filter. We used the altimetry product provided by the Hydrographic and Oceanographic Service of the French Navy (SHOM) at a 25 m^2^ resolution to identify these erroneous locations and discard them. Positions associated with a travel speed of over 10 km/hr were discarded (8%), and also those associated with location class Z (0.1%), that were considered insufficiently accurate.

## 3 RESULTS

### 3.1 Genetic diversity, population structure, and Mixed Stock Analyses

**Figure 1:**
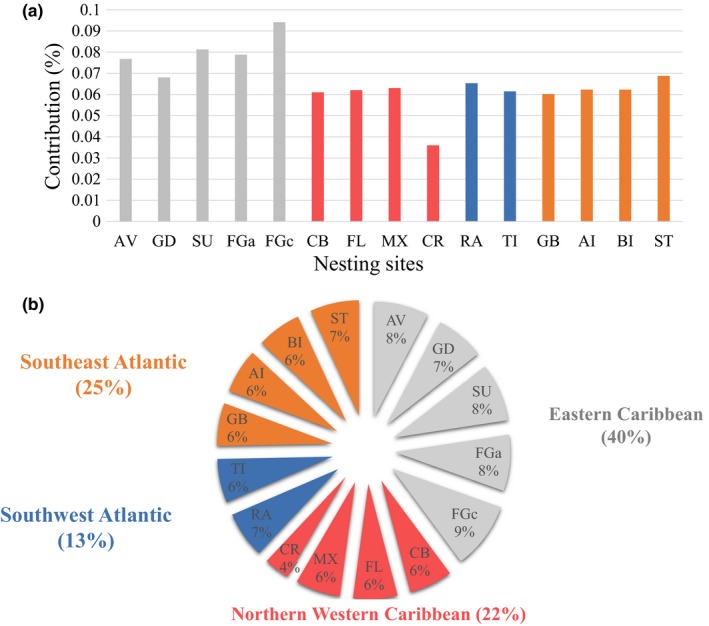
(a) Histogram and (b) pie chart of the contribution of nesting sites stock to feeding ground of Martinique (MT). In (b) each contribution was converted into percentage, and the four colors refer to the four regions: Eastern Caribbean in gray, Northern Western Caribbean in red, Southwest Atlantic in blue and Southeast Atlantic in orange. The associated proportions of the four regions were added in parentheses. Abbreviations refer as follows: AI: Ascension Island; AV: Aves Island; BI: Bioko; CB: Cuba; CR: Costa Rica; FGa, West side of French Guiana: French Guiana Awala; FGc, east side of French Guiana: French Guiana Cayenne; FL: Florida; GB: Guinea‐Bissau; GD: Guadeloupe; MX: Mexico; RA: Rocas Atoll; ST: Sao Tome; SU: Suriname; TI: Trindade Island

A total of 40 green turtles of the mtDNA control region in the feeding ground of Martinique (hereafter MT) and from 15 nesting populations have been used (Table 1). The results are presented in Figure 1. A total of six haplotypes were identified in MT feeding ground (CM‐A1, CM‐A3, CM‐A5, CM‐A8, CM‐A9 and CM‐A50). Five haplotypes have been excluded from the analyses: CM‐A50 was only represented by one individual in MT and one in the Ascension Island (AI) nesting site, and CM‐A4, CM‐A6, CM‐10, and CM‐12 were not represented in feeding ground MT (Table 1). Gene (Hs) and nucleotide (*π*) diversities were 0.674 ± 0.063 and 0.005 ± 0.003 for Martinique, and the closest nesting sites show diversity of 0.476 ± 0.128 and 0.002 ± 0.001 for Cayenne (French Guiana), 0.239 ± 0.113 and 0.000 ± 0.000 for Awala (French Guiana), and 0.0625 ± 0.058 and 0.000 ± 0.000 for Suriname. Pairwise tests (Tamura and Nei distance method) among feeding ground MT and nesting sites revealed significant structure. Genetic structure was the strongest between Martinique and FG Awala (*F*_st_ = 0.225; *p* < 0.005) and Martinique and Suriname (*F*_st_ = 0.241; *p* < 0.005), while the genetic structure between Martinique and FG Cayenne appeared to be the lowest (*F*_st_ = 0.120; *p* < 0.005). Global exact tests of differentiation showed low but significant structure (exact *p* < 0.005) between Cayenne and Awala (*F*_st_ = 0.033; *p* < 0.005), and between Cayenne and Suriname (*F*_st_ = 0.032; *p* < 0.005). On the other hand, no structure was found between Awala and Suriname.

**Table 1:** Haplotypes and haplotype frequency of the Martinique feeding ground (MT, shaded line) investigated and nesting sites considered in this study

Haplotype	CM‐A1	CM‐A3	CM‐A4	CM‐A5	CM‐A6	CM‐A8	CM‐A9	CM‐A10	CM‐A12	CM‐A50
MT	4	5	0	21	0	8	1	0	0	1
AV*	0	3	0	27	0	0	0	0	0	0
GD*	0	1	0	35	0	0	0	0	0	0
SU	0	0	0	31	0	1	0	0	0	0
FGc	0	0	0	17	1	2	0	1	1	0
FGa	0	0	1	21	0	0	0	0	0	0
CB*	3	16	0	0	0	0	0	0	0	0
FL*	11	12	0	0	0	0	0	0	0	0
MX*	7	5	0	1	0	0	0	0	0	0
CR*	0	95	0	32	0	0	0	0	0	0
RA*	0	0	0	0	0	36	7	0	0	0
TI*	0	0	0	0	0	67	19	0	0	0
GB*	0	0	0	0	0	70	0	0	0	0
AI*	0	0	0	0	0	204	9	0	0	1
BI*	0	0	0	0	0	45	0	0	0	0
ST*	0	0	0	1	0	13	0	0	0	0

Note

AI: Ascension Island; AV: Aves Island; BI: Bioko; CB: Cuba; CR: Costa Rica; FGa: French Guiana Awala; FGc: French Guiana Cayenne; FL: Florida; GB: Guinea‐Bissau; GD: Guadeloupe; MT: Martinique; MX: Mexico; RA: Rocas Atoll; ST: Sao Tome; SU: Suriname; TI: Trindade Island.

Nesting sites from those listed in Jordao et al. (2017) are marked with an asterisk (*).

Shrink factors estimated with the Gelman and Rubin (1992) diagnostic revealed values close to one for all stocks and confirm a convergence strength of chains. Many‐to‐one analyses indicated French Guiana (both Awala and Cayenne), Suriname, and Aves Island as the most important source population to MT population, and more largely the Eastern Caribbean, accounting for 40% of the contribution to the foraging ground of Martinique (Figure 1).

### 3.2 Tag performance and morphometric data

Among the 31 juvenile turtles tracked between 2013 and 2017, 21 remained in Martinique waters whereas the 10 other turtles left Martinique between June (*n* = 6, #149691, #149692, #149693, #149694, #149696, and #149697) and October (*n* = 4, #150122, #164547, #164548, and #45811) to perform a migration across the Caribbean–Atlantic region (#45811, #149691, #149692, #149693, #149694, #149696, #149697, #150122, #164547, and #164548). The tag instruments transmitted on average (mean ± *SD*) 515 ± 349 locations (range: 142 to 886, #149691 vs. #164548, respectively). The length of these migrant individuals varied from 78.5 to 93 cm (mean ± *SD*: 85.9 ± 3.8 cm, #149697 vs. #48511, respectively) and their body mass from 61.6 to 91.6 kg (mean ± *SD*: 81 ± 9.3 kg, #149697 vs. #48511, respectively). The 21 resident individuals were significantly smaller (mean CCL ± *SD*: 75.5 ± 10.5 cm, Kruskal–Wallis rank sum test, *p* < 0.05) than the 10 migrant turtles (85.9 ± 3.8 cm). The tracking duration was on average 166 ± 79 days, and the total distance travelled ranged from 1,370 km (#149697) to 7,821 km (#149696)—see Table 2.

**Table 2:** Summary of the horizontal movements of the 10 juvenile green turtles tracked from Martinique developmental habitat

PTT	Start date	End date	Nloc	Duration (days)	Distance (km)	Speed (km/hr)	CCL (cm)	Body mass (kg)
150122	14/10/2015	31/07/2016	306	291	5,094	1.4 ± 1.2	82	66.4
149691	03/06/2015	24/07/2015	142	51	2,649	1.7 ± 1.6	81	67.4
149692	03/06/2015	04/12/2015	259	184	4,213	1.4 ± 1.3	86	80.4
149693	02/06/2015	14/10/2015	171	134	2,645	1.1 ± 1.4	83	71.5
149694	01/06/2015	18/08/2015	364	78	3,098	2.2 ± 1.4	84	75.4
149696	03/06/2015	20/12/2015	429	200	7,821	2.2 ± 1.2	88	81.4
149697	02/06/2015	16/03/2016	567	288	1,370	0.6 ± 0.9	78.5	61.6
164547	27/10/2016	21/03/2017	845	145	5,844	2.1 ± 1.2	88	73.8
164548	25/10/2016	22/03/2017	886	148	6,104	2.0 ± 1.3	88.5	77.6
45811	26/10/2017	10/03/2018	1,186	135	5,108	1.9 ± 1.2	93	91.6
			*515* ± *349*	*166* ± *79*	*4,394* ± *1,964*	*1.6* ± *1.2*	*85* *.* *9* ± *3.8*	*81* ± *9.3*

Note

CCL: curved carapace length; Nloc: number of locations analyzed in the study; PTT: Platform Terminal Transmitter.

The numbers in italics are the mean + *SD*.

### 3.3 Migratory routes

**Figure 2:**
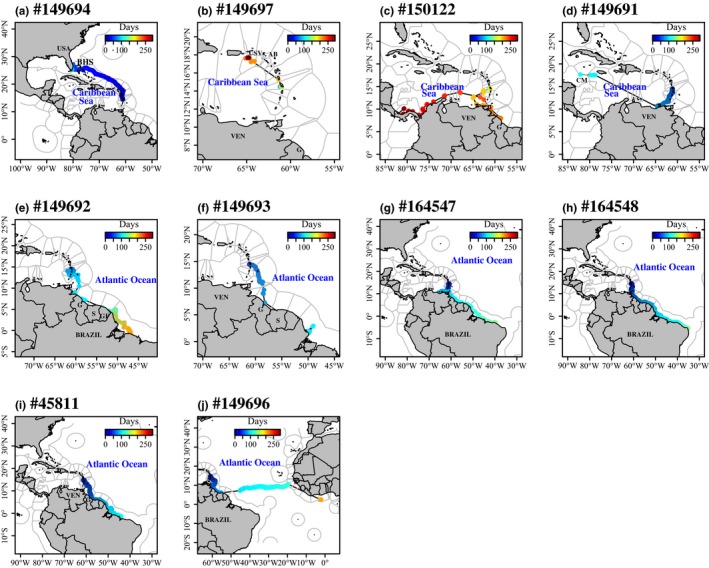
Tracks (black lines) and locations (in color) of the 10 juvenile green turtles that performed developmental migration from Martinique. The color bar indicates the time elapsed (in days) since tag deployment. The black lines with no dots refer to missing locations due to transmission issues. The gray contours refer to the Exclusive Economic Zones crossed during migration, namely AB: Antigua and Barbuda; BHS: the Bahamas; CM: the Cayman Islands; G: Guyana; GB: Guinea‐Bissau; GN: Ghana; S: Suriname; USA: United States; USV: United States Virgin Islands; VEN: Venezuela

The 10 individuals headed in six different directions, with 50% to the North Brazilian coast (*n* = 5), 30% to the Caribbean (*n* = 3), 10% to United States (*n* = 1) and 10% to Africa (*n* = 1)—see Figure 2. Only two juvenile turtles reached a possible conclusive destination (#149694 and #149697, Figure 2a,b). Turtle #149694 left Martinique immediately and headed north‐westward to reach Florida, which could be considered her final destination as she remained there for at least one week (Figure 2a). Then, she reached the US Virgin Islands, where she remained for several weeks. Turtle #149697 remained close to Martinique Island during approximately three months before traveling toward Antigua and Barbuda (Figure 2b).

Turtles #150122 and #149691 migrated south‐westward (Figure 2c,d). After stopping emitting for a month (probably due to transmission issues or the short surfacing behavior of green turtles), the tag #149691 transmitted again, indicating that the individual was swimming along the coast of Jamaica as far as until the Caiman Islands (Figure 2d), while the tag #150122 followed the Colombian coast toward Costa Rica (Figure 2c).

**Figure 3:**
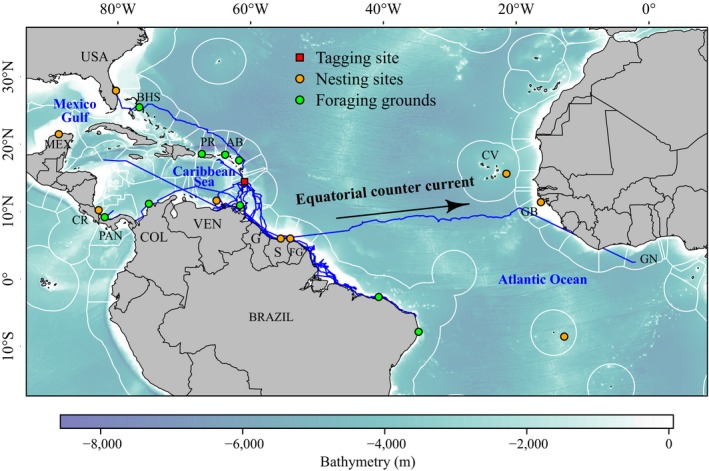
Tracks of the 10 juvenile green turtles in relation to known habitats of adult green turtles across the Caribbean–Atlantic region, that is, nesting sites (in orange) and foraging grounds (in green). The white contours refer to the Exclusive Economic Zones: AB: Antigua and Barbuda; BHS: the Bahamas; CM: the Cayman Islands; COL: Colombia; CR: Costa Rica; CV: Cape Verde; G: Guyana: GB: Guinea‐Bissau; GN: Ghana; MEX: Mexico; PAN: Panama; PR: Puerto Rico; S: Suriname; USA: United States; USV: United States Virgin Islands; VEN: Venezuela. The bathymetry was extracted from GEBCO (30‐arc‐second 1 km grid)

Turtles #149692, #149693, #164547, #164548, and #45811 headed in the same direction, south‐eastward to reach the Guyana coast (Figure 2e–i). After remaining around the French West Indies for approximately 50 days, the individuals #149692 and #149693 stopped transmitting over the Amazon River plume. The three turtles (#164547, #164548, and #45811) reached the known foraging grounds off the State of Ceará in Brazil (Figure 2g–i). During the tracking duration, the five individuals crossed around 25 territorial waters across the Caribbean–Atlantic region (Figures 2 and 3). After following the same direction as the five preceding turtles, the individual #149696 left the Surinamese coast to head eastward toward the African coast, probably benefiting from the Equatorial Counter‐Current (Figures 2j and 3).

## 4 DISCUSSION

This study made it possible to assess the natal origins of juvenile green turtles inhabiting Martinique waters, and to identify their connecting paths between the developmental habitat of Martinique and already known habitats as inhabited by adult green turtles across the Caribbean–Atlantic region. Prior to our study, there was very little information on the movements of juvenile green turtles in the Caribbean.

Our results show that the genetic diversity of the Martinique juvenile turtles is high. Mixed stock analysis enables to show the contribution of different nesting sites to a feeding ground (Jordao et al., 2017; Okuyama & Bolker, 2005). The mitochondrial CMA‐5 haplotype that dominates in the Caribbean is the most prevalent (Bass et al., 2006; Bjorndal et al., 2005; Lahanas et al., 1998). Other haplotypes from other regions were found, such as CMA‐8 which is widely reported in southern Atlantic rookeries (Jordao et al., 2017) and in feeding grounds in the Caribbean and Atlantic (Lahanas et al., 1998; Luke et al., 2003). Our results confirm that those juveniles are descendant from females laying on several Caribbean and Atlantic nesting sites, mostly from the Surinamese and French Guianese beaches, but also from other western Caribbean sites, and from more southern Brazilian nesting beaches. Several variables may influence the connectivity between feeding and nesting sites, such as the geographic distance, the number of nesting females, the oceanic currents, and the historical climatic changes (Bass & Witzell, 2000; Lahanas et al., 1998; Luke et al., 2003; Naro‐Maciel et al., 2012). Unfortunately, our results do not clarify either juveniles migrate to Martinique via passive dispersal (via the Guiana current or the North Brazilian current) or an active migration from nesting beaches.

The average size of our migrant turtles was larger than those found by Meylan, Meylan, and Gray (2011) (mean CCL in Martinique: 85.9 cm vs. 81.9 cm), who performed a series of laparoscopies on juvenile green turtles in Panama to determine the minimum size at sexual maturity. Although the turtles tracked from Martinique waters were smaller (CCL range: 78.5–93 cm) than the reproductive females observed in French Guiana (CCL range: 103–133 cm, Chambault, de Thoisy, Kelle, et al., 2016), Costa Rica (CCL range: 95.5–110.9 cm, Troëng, Evans, Harrison, & Lagueux, 2005), Ascension Island (CCL range: 100–130 cm, Hays, Broderick, Glen, & Godley, 2002), and Guinea‐Bissau (CCL range: 92–103 cm, Godley, Almeida, et al., 2003; Godley, Lima, et al., 2003), this suggests that our migrant individuals are close to reach sexual maturity. These turtles might therefore initiate their migration, either to discover new developmental habitats (Carr, Carr, & Meylan, 1978) or to directly reach adult habitats (foraging grounds or breeding sites). Among the 31 green turtles tracked from Martinique, 21 remained in the shallow waters of Martinique. The smaller size of these 21 resident turtles (mean CCL: 75.5 ± 10.5 cm) compared to the size of the migrants (85.9 ± 3.8 cm) suggested a threshold beyond which individuals start migrating, therefore reinforcing the assumption that the migrant turtles may be close to sexual maturity.

The multiple origins of these juvenile turtles in the Caribbean–Atlantic region have been confirmed by the tracks of migrant turtles that headed toward adult habitats (natal beaches and foraging grounds) (see Figure 3). Similar behavior was observed for some juvenile green turtles in the Indian Ocean which had shorter CCL than our migrant individuals (73.5 ± 4.9 cm vs. 85.9 ± 3.8 cm), but successfully reached adult foraging areas (Pelletier, Roos, & Ciccione, 2003). Although in the present study most of the tags did not transmit locations until the turtles reached their final destination, the trajectories recorded confirm our assumption on the link between adult habitats (foraging or breeding sites) and the developmental habitat of Martinique.

Two individuals (#149694 and #149697) headed north‐westward, passing by several adult foraging grounds (US Virgin Islands, Antigua and Barbuda, Puerto Rico, Dominican Republic and Bahamas). Turtle #149697 performed a short migration and remained for several weeks in the US Virgin Islands, known to be a famous foraging ground for both juvenile and adult green turtles. They feed there on *Thalassia testudinum*, the same species that is found in Martinique (Ogden, Robinson, Whitlock, Daganhardt, & Cebula, 1983). Despite the availability of different foraging grounds and nesting sites along her route, turtle #149694 targeted one site located in Florida rather than exploring new developmental habitats: after crossing the many nesting sites located north of West Palm Beach (Roberts, Collins, Paxton, Hardy, & Downs, 2014; Shamblin et al., 2014), and the juvenile reef habitats (Stadler, Salmon, & Roberts, 2014), this turtle ended its migration in Biscayne Bay which hosts different communities of seagrasses and macroalgae, as well as many megafauna groups, including sea turtles (Lirman et al., 2014). The seagrasses and macroalgae taxa sampled in Biscayne Bay are also similar to those found in the French West Indies, that is, *Halodule*, *Syringodium,* and *Thalassia *(Chevallier et al., 2016; DEAL Guyane & Agence des Aires Marines Protégées, 2013), and they are particularly abundant where this individual spent most of its time, that is, near Manatee Bay (Lirman et al., 2014).

Two other turtles migrated south‐westward (#149691 and #150122), toward the Venezuelan and Panama coasts, respectively. After one month with no location, the tag of turtle #149691 started transmitting again once the individual had crossed the Venezuelan coast, indicating that this turtle was heading for Honduras, possibly targeting either the Honduras nesting site or other adult habitats located in the Gulf of Mexico. Turtle #150122 followed the Panama coast, targeting either the Panama foraging ground or the Costa Rica nesting site described by Troëng and Rankin (2005)—see Figure 3.

Conversely, turtle #149696 used prevailing currents (during the second part of the migration) to reach her destination. This individual left the French Guianan coast to perform the first transatlantic migration ever recorded in juvenile green turtles, swimming through the Equatorial Counter‐Current (Figure 3, Fonseca, Goni, Johns, & Campos, 2004) toward Guinea‐Bissau, and then Ghana. Guinea‐Bissau is the most important nesting site for green turtles along the African coast (Catry et al., 2002), and is also a major contributor to the genetic diversity of the West Atlantic foraging grounds (Jordao et al., 2015). The probability that this turtle ended its journey either in the nesting site of Guinea‐Bissau or Cape Verde is reinforced by the simulations performed by Putman and Naro‐Maciel (2013), who demonstrated that the dispersal east of 14°W accounted for 5%–65% of the simulated turtles found in the Caribbean and North American foraging grounds after 5 years of simulations (Putman & Naro‐Maciel, 2013).

Fifty percent of the tracked turtles (#149692, #149693, #164547, #164548, and #45811) headed south‐eastward, and reached the same Brazilian foraging grounds used by both adult green turtles coming from French Guiana (Baudouin et al., 2015; Chambault et al., 2015) and juvenile green turtles (Godley, Almeida, et al., 2003; Godley, Lima, et al., 2003; Lima, Lagueux, Castro, & Marcovaldi, 1999; Lum, Lima, & Santos, 1998). These results confirm the significant contribution of the Suriname–French Guianan and the southern Brazilian nesting sites to genetic diversity found in the Brazilian feeding grounds (Jordao et al., 2015, 2017). For turtles originating from the Suriname‐French Guiana nesting beaches, there will be a need for an active swimming behavior toward specific destinations when comparing the tracks of these five turtles with the oceanic circulation. Similarly to the behavior observed in adult green turtles originating from French Guiana (Chambault et al., 2015), these juvenile individual swam against both the north‐westward flowing Guiana current and North Brazilian current (Baklouti et al., 2007; Fratantoni & Glickson, 2002).

For adequate geographic and ecological scaling, species conservation plans should take into account the genetic structure and demographic history of populations (Lande, 1988). Genetic clades may vary according to gender, age, and bioecological function (e.g., feeding vs. breeding) taken into account (Jordao et al., 2017). The genetic structure of *Chelonia mydas* in the West Atlantic revealed movements of individuals between northern (this study) and southern feeding areas (Jordao et al., 2017), and northern nesting rookeries, and reinforces the conservation importance of regional corridors.

The extensive movements of the 10 migrant turtles evidenced the crossing of international waters and more than 25 exclusive economic zones. The multidirectional migrations performed by these 10 individuals, reinforced therefore the need for an international cooperative network to ensure the conservation of future breeders in this endangered species. Our result is also in accordance with the genetically mixed stock of green turtles inhabiting common developmental habitats but originating from diverse natal beaches dispersed across the Caribbean (Bass & Witzell, 2000; Jordao et al., 2015; Luke et al., 2003). Using the same genetic analyses as those already carried out for loggerhead (Engstrom, Meylan, & Meylan, 2002), hawksbill (Meylan, 1990), and green turtles (Bass et al., 2006; Luke et al., 2003), we will be able to find the natal origins of these juvenile turtles and confirm whether they are heading for adult habitats or rather exploring new developmental habitat until they migrate toward adult habitats. Since this population also benefits of a long‐term follow up via capture and tagging (PIT) of individuals in Martinique foraging sites, the recapture of tagged turtles in the multiple nesting sites spread across the Atlantic will soon make it possible to link the developmental habitat of Martinique to natal nesting beaches (i.e., French Guiana and Suriname, and likely others). In order to support the conservation of this endangered species at multiple scales and stages, that is, developmental habitat, foraging areas, and reproduction sites, it is crucial to identify the key areas used by juvenile green turtles to ensure the protection of these future breeders, via the track of additional individuals in the different Caribbean islands. Data from these studies will consolidate decision making for the application of urgent conservation measures in this extensive area composed of multiple jurisdictional waters. This study provides up‐to‐date and useful genetic and tracking data that could more accurately support the revision of the geographic limits of Regional Management Units (Wallace et al., 2010).

## CONFLICT OF INTEREST

None declared.

## AUTHOR CONTRIBUTIONS

PC performed the data analysis and wrote the manuscript. DC designed the experiment, collected the data, and supervised the analysis. PC, JM, MB, DE, JG, GH, JM, FV, CB, ES, BG, EDD, SR, NL, FL, CF, NA, CG, RB, LT, MD, MB, AP, FF, PQ, TW, JPA, NC, AB, CM, TM, LR, NC, BC, CB, YLM, and OP participated in the field effort. PC, BdT, MH, and DC assisted with organizing the data and analysis and interpretation of the results. All the authors shared the responsibility for contributing to the final version of the manuscript.

## DATA ACCESSIBILITY

DNA sequences were deposited to GenBank, accession numbers range from MK060180 to MK060219.
